# Supplementary material for: Phosphaturic Mesenchymal Tumors: Rethinking the Clinical Diagnosis and Surgical Treatment
Source: J Clin Med. 2022 Dec 29;12(1):252. doi: 10.3390/jcm12010252 (PMC9821698; doi:10.3390/jcm12010252)
Supplement: Supplementary file 1 [file jcm-12-00252-s001.zip › jcm-2043256-supplementary.pdf]

Supplemental Table S1: General information

Patient	Gender	Age	Duration of disease (month)	Location	Tumor size(cm)	Pathological fracture	Treatment before diagnosis	Therapeutic method	Duration of Follow-up (month)	Preoperative MSTS score	Postoperative MSTS score	Preoperative VAS score	Postoperative VAS score	Recurrence or metastasis
1	M	60	24	humerus	5.3	yes	surgery	MAAEC	43	17	28	4	0	no
2	M	40	16	soft tissue of shoulder	2.5	no	medication	RR	55	13	30	6	0	no
3	M	47	34	femur	1.8	no	medication	MAAEC	27	15	29	5	0	no
4	F	27	32	femur	4.5	yes	surgery	RR	34	13	28	6	1	no
5	F	57	20	tibia	3.8	yes	medication	MAAEC	73	10	28	7	0	no
6	M	32	13	soft tissue of thigh	3.4	yes	medication	RR	42	18	30	4	0	no
7	F	55	18	ilium	2.6	yes	medication	MAAEC	29	16	29	5	1	no
8	M	29	9	femur	1.7	no	surgery	MAAEC	37	21	30	4	0	no
9	F	44	13	ilium	5.5	yes	medication	MAAEC	43	17	28	5	1	no
10	F	59	35	femur	4.2	yes	surgery	MAAEC	57	16	29	6	1	no
11	F	12	25	scapula	6.3	yes	medication	RR	64	14	30	5	0	no
12	F	38	15	femur	4.5	yes	surgery	MAAEC	50	14	29	6	1	no

MAAEC,microwave ablation-assisted extended curettage; RR,radical resection

Supplemental Table S2: Biochemical examination

**Patient**	**Phosphorus**				**Calcium**		**AKP**			**PTH**		**TPINP**	
	Preop	Postop					Preop	Postop					
		3day	7day	14day	Preop	Postop		3day	14day	Preop	Postop	Preop	Postop
**1**	0.31	0.66	0.97	1.21	2.24	2.14	234.1	154.6	76.8	46.05	50.6	164.9	56.9
**2**	0.54	0.75	1.12	1.43	2.14	2.16	323.5	218.3	113.5	60.7	53.5	185.5	86.2
**3**	0.42	0.72	1.04	1.14	2.42	2.33	218.4	98.6	58.6	82.55	57.3	81.53	65.8
**4**	0.5	0.64	0.92	1.25	2.24	2.52	164.9	76.5	73.5	511	73.5	70.29	60.4
**5**	0.35	0.69	0.86	1.45	2.42	2.16	235.6	121.5	86.4	53.7	47.6	31.55	44.6
**6**	0.36	0.87	1.18	1.35	2.37	2.24	575	208.7	113.4	77.4	55.4	138.9	87.3
**7**	0.59	0.88	1.01	1.34	2.09	2.01	337.4	235.8	120.6	68.9	71.4	124.7	90.8
**8**	0.42	0.62	0.87	1.23	2.15	2.15	344.6	189.7	89.7	52.02	48.7	56.8	50.4
**9**	0.38	0.67	0.93	1.16	2.24	2.05	143.4	90.5	68.3	59.3	57	66.9	53.8
**10**	0.26	0.47	1.02	1.38	2.11	2.08	191.5	121.5	110.4	81.11	69.2	30.63	35.6
**11**	0.45	0.78	1.04	1.45	2.15	2.21	431.4	223.6	123	74.9	66.3	143.8	75.5
**12**	0.41	0.67	1.07	1.31	2.23	2.25	216.5	154.1	90.8	68.3	60.5	121.6	86.5
**Normal range**	***0.86-1.78mmol/L***				***2.00-2.60mmol/L***		***45.0-125.0U/L***			***15-65pg/ml***		***9.06-76.24ng/ml***	
